# Impact of neoadjuvant intensity-modulated radiation therapy on borderline resectable pancreatic cancer with arterial abutment; a prospective, open-label, phase II study in a single institution

**DOI:** 10.1186/s12885-022-09244-6

**Published:** 2022-01-29

**Authors:** Toshihiko Masui, Kazuyuki Nagai, Takayuki Anazawa, Asahi Sato, Yuichiro Uchida, Kenzo Nakano, Akitada Yogo, Akihiro Kaneda, Naoto Nakamura, Michio Yoshimura, Takashi Mizowaki, Norimitsu Uza, Akihisa Fukuda, Shigemi Matsumoto, Masashi Kanai, Hiroyoshi Isoda, Masaki Mizumoto, Satoru Seo, Koichiro Hata, Kojiro Taura, Yoshiya Kawaguchi, Kyoichi Takaori, Shinji Uemoto, Etsuro Hatano

**Affiliations:** 1 Department of Surgery, Graduate School of Medicine, Kyoto University, Kyoto, Japan; 2 Department of Radiation Oncology and Image-Applied Therapy, Graduate School of Medicine, Kyoto University, Kyoto, Japan; 3 Department of Gastroenterology and Hepatology, Kyoto University, Kyoto, Japan; 4 Department of Real World Data Research and Development, Graduate School of Medicine, Kyoto University, Kyoto, Japan; 5 Department of Clinical Oncology, Kyoto University, Kyoto, Japan; 6 Department of Diagnostic Imaging and Nuclear Medicine, Kyoto University, Kyoto, Japan

**Keywords:** Neoadjuvant therapy, Pancreatic cancer, Intensity-Modulated Radiotherapy, Surgery

## Abstract

**Background:**

Borderline resectable pancreatic cancer (BRPC) is a category of pancreatic cancer that is anatomically widely spread, and curative resection is uncommon with upfront surgery. Intensity-modulated radiation therapy (IMRT) is a form of radiation therapy that delivers precise radiation to a tumor while minimizing the dose to surrounding normal tissues. Here, we conducted a phase 2 study to estimate the curability and efficacy of neoadjuvant chemoradiotherapy using IMRT (NACIMRT) for patients with BRPC with arterial abutment (BRPC-A).

**Methods:**

A total of 49 BRPC-A patients were enrolled in this study and were treated at our hospital according to the study protocol between June 2013 and March 2021. The primary endpoint was microscopically margin-negative resection (R0) rates and we subsequently analyzed safety, histological effect of the treatment as well as survivals among patients with NACIMRT.

**Results:**

Twenty-nine patients (59.2%) received pancreatectomy after NACIMRT. The R0 rate in resection patients was 93.1% and that in the whole cohort was 55.1%. No mortality was encountered. Local therapeutic effects as assessed by Evans classification showed good therapeutic effect (Grade 1, 3.4%; Grade 2a, 31.0%; Grade 2b, 48.3%; Grade 3, 3.4%; Grade 4, 3.4%). Median disease-free survival was 15.5 months. Median overall survival in the whole cohort was 35.1 months. The only independent prognostic pre-NACIMRT factor identified was serum carbohydrate antigen 19–9 (CA19-9) > 400 U/ml before NACIMRT.

**Conclusions:**

NACIMRT showed preferable outcome without significant operative morbidity for BRPC-A patients. NACIMRT contributes to good local tumor control, but a high initial serum CA19-9 implies poor prognosis even after neoadjuvant treatment.

**Trial Registration:**

UMIN-CTR Clinical Trial: https://upload.umin.ac.jp/cgi-open-bin/ctr_e/ctr_view.cgi?recptno=R000011776

Registration number: UMIN000010113.

Date of first registration: 01/03/2013,

## Background

Pancreatic cancer is one of the most poorly prognosed malignancies, with high mortality rates worldwide [1]. This disease is the fourth leading cause of cancer deaths in Japan, and its incidence is rising with the aging of the population [2]. For pancreatic cancer without metastasis, surgical resection offers the highest cure rate. However, curative resection is sometimes difficult if the tumor is overly close to vital arteries or veins. The National Comprehensive Cancer Network (NCCN) has proposed the category of “borderline resectable pancreatic cancer” (BRPC) for such tumors [3]. BRPC is defined as a tumor meeting any of the following criteria: BRPC-A; 1) focal tumor abutment (in contact with ≤ 180° of vessel circumference) of the superior mesenteric artery (SMA) or of the celiac axis (CA); 2) encasement of common hepatic artery (CHA) but not to the CA or proper hepatic artery (PHA); or BRPC-V; 3) involvement of the superior mesenteric vein (SMV)/ portal vein (PV) with abutment more than180°. Given these definitions, BRPC-A represents a particularly difficult entity when trying to achieve curative resection [4]. Recently, neoadjuvant therapy with FOLFIRINOX [5] or gemcitabine plus radiotherapy [6, 7] for BRPC patients has shown favorable microscopically margin-negative resection (R0) rates, but the contributions to survival have remained contentious. Similarly, our previous phase 2 study with gemcitabine and S-1 showed better R0 rates compared to upfront surgery, but failed to show any survival advantage for those patients [8]. Because of the high rates of R0 after neoadjuvant therapy, patients with BRPC-A might benefit most from neoadjuvant therapy with additional radiation.

Intensity-modulated radiation therapy (IMRT) is a radiotherapeutic technique that allows higher radiation doses to be focused to regions while minimizing the dose to normal tissue. The advantage of IMRT over conventional radiation therapy is that it maximizes the effect on the target tissue and reduces the toxicity to the surrounding normal tissue. [9, 10]. Although IMRT has been used for other tumors such as prostate cancer [11, 12] and nasopharyngeal carcinoma [13, 14], few data has been accumulated on its efficacy in treating patients with pancreatic cancer, and even less on survival outcomes [15, 16], because the target tissue shifts with respiration, making it difficult to irradiate the tissue accurately [17]. We have reported a favorable outcome of IMRT to patients with non-metastatic locally advanced pancreatic cancer [18].

Here, we have conducted a prospective phase 2 study for BRPC-A patients to analyze the impact of neoadjuvant chemoradiotherapy using IMRT (NACIMRT) with gemcitabine on surgical curability and survival.

## Methods

### Study design and Patients

This study was conducted as a prospective phase II study of neoadjuvant treatment with IMRT plus gemcitabine (UMIN000010113) for BRPC-A patients. The primary endpoint was the R0 rate to evaluate the effect of IMRT (total dose, 42 Gy) with gemcitabine as neoadjuvant therapy for BRPC-A. All patients with pancreatic tumors classified as BRPC-A according to NCCN 2009 guidelines diagnosed at our hospital between June 2013 and March 2021 and who provided consent were enrolled to this study. The extent of tumor involvement as BRPC-A was assessed from multidetector-row computed tomography (MDCT) using a multiphase contrast-enhanced technique and evaluated by a multidisciplinary team for pancreatic cancer comprising doctors from the Department of Surgery, Department of Gastroenterology, Department of Radiation Oncology, Department of Clinical Oncology and Department of Diagnostic Imaging.

Inclusion criteria for BRPC-A pancreatic cancer in this study were as follows. In brief, with the contrast-enhanced MDCT, patients showing tumor abutment with the SMA at =  < 180 degree of the vessel circumference, or tumor abutment with the CHA allowing complete resection were defined as BRPC-A. Tumors with abutment of the CA but not to the aorta that could be completely resected by distal pancreatectomy with celiac axis resection were also categorized as BRPC-A. Other inclusion criteria were as follows: histologically confirmed pancreatic ductal adenocarcinoma; age > 20 but < 80 years; and ECOG performance status of 0 or 1, no distant metastasis in the thorax, abdomen or pelvis on dynamic contrast-enhanced MDCT, on positron emission tomography with 2-deoxy-2-[^18^F]fluoro-D-glucose (FDG-PET) and on magnetic resonance imaging (MRI) with contrast medium of gadolinium-ethoxybenzyl diethylenetriaminepentaacetic acid (EOB-MRI), no pre-treatment for current pancreatic cancer, and no hematological dysfunction or that of the main organs.

Exclusion criteria were as follows: interstitial pneumonitis; history of irradiation to the upper abdomen; serious comorbidities (heart failure, renal failure, liver failure, bleeding peptic ulcer, intestinal paralysis, intestinal obstruction, uncontrolled diabetes); moderate or severe ascites or pleural effusion; history of active cancer (concurrent multiple cancers or heterogeneous multiple cancers with a disease-free interval of less than 3 years); or expectant or nursing women. The Ethics Committee of Kyoto University approved this study, and each patient gave informed consent prior to participation.

### Neoadjuvant chemoradiotherapy

**Fig. 1:**
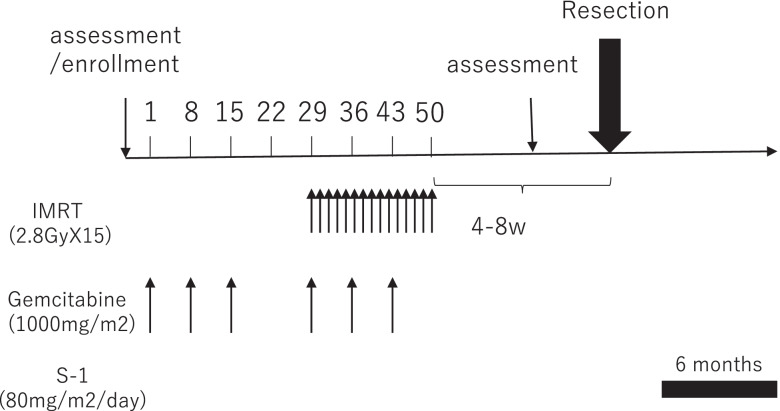
The treatment schedule consisted of induction chemotherapy with gemcitabine (1000 mg/m^2^), preoperative IMRT at 42 Gy (2.8 Gy/day, 5 times a week, 15 fractions in total), and intravenous gemcitabine administered over 30 min on days 22, 29, and 36. Radiological re-assessment was performed 4–6 weeks after the final irradiation

The treatment for NACIMRT is presented in Fig. 1. After the tumor was diagnosed histologically as adenocarcinoma by endoscopic ultrasonographic fine needle aspiration, patients were initially administered gemcitabine 3 times (days 1, 8, and 22) at a dose of 1000 mg/m^2^ before chemoradiotherapy. On starting radiotherapy, gemcitabine was administered at a dose of 1000 mg/m^2^ (days 1, 8, and 22) concurrent with IMRT. When grade 4 or worse neutrocytopenia or thrombocytopenia occurred, chemotherapy was stopped for a week. For IMRT planning, gross tumor volume (GTV) included the pancreatic tumor and any lymph nodes > 1 cm in diameter. Clinical target volume (CTV) included the celiac and para-aortic lymph node basins, in addition to the GTV plus a 5-mm margin, according to our institutional contouring guidelines. Organs at risk were the liver, stomach, duodenum, small intestine, colon and kidneys, as well as the spinal cord, and were delineated on expiratory-phase CT. The planning target volume (PTV) was defined as the CTV with a 5-mm margin in all directions. The prescription dose of 42 Gy administered in 15 fractions was specified as D95 (the dose covering 95% of the target structure) to PTV-boost. PTV-boost is a volume that subtracted the stomach plus 10-mm, and the duodenum plus 5-mm margins from the PTV. IMRT was used to generate optimized treatment plans for each patient. Breath-hold method was adopted for the management of tumor respiratory motion and daily cone beam CT before each treatment was used to determine the daily set-up errors. Radiation treatment was delivered with volumetric modulated arc therapy techniques.

### Resection and adjuvant chemotherapy

Patients were evaluated for resection within 4 weeks after neoadjuvant therapy using MDCT, EOB-MRI, and FDG-PET and were examined by our multidisciplinary pancreatic cancer treatment team. In the absence of clear technical unresectability, resection was attempted between 4 and 8 weeks after finishing neoadjuvant radiotherapy. Pancreaticoduodenectomy, distal or total pancreatectomy (and resection of any involved tissues) was performed according to the tumor location. Operative findings, surgical complications, and histopathology were recorded. S-1 at a dose of 80 mg/m^2^/day was administered on days 1–28 of a 42-day cycle for 6 months as adjuvant chemotherapy, starting 4–8 weeks after resection.

### Assessment

Resection margins were determined as positive (R1) if malignant cells were observed at the surface of the resected specimen (0-mm margin rule), the plexus around the SMA or CHA, duodenum, bile duct, or retroperitoneal tissue. If vein was concomitantly resected, the vein margin was examined additionally.

Follow-up data were examined on medical records up to August 2021. Patients’ status was evaluated by contrast-enhanced CT every 3 months for the first 2 years, then every 6 months thereafter. The first site of disease recurrence was defined as follows: A new low-density mass in the peripancreatic and mesenteric root area was considered a locoregional recurrence. For locoregional failure-free interval (LFFI) analysis, locoregional failure was only the event of interest and was defined as the appearance of tumors in the region of the resected pancreatic bed and root of the mesentery. For distant metastasis-free interval (DMFI) analysis, distant metastatic failure was the event of interest and was defined as a new low-density region in the liver or lungs as well as new ascites on ultrasonography or CT, subsequently confirmed by cytology as peritoneal dissemination. Disease-free survival (DFS) was calculated as the time from the date of surgery to that of initial recurrence. Overall survival (OS) was calculated as the time from the date of initial treatment to that of death. Tumor length was estimated based on the contrast-enhanced CT image before treatment and on the resected specimen. Toxicity events were recorded using the Common Terminology Criteria for Adverse Events (CTCAE version 4.0; https://ctep.cancer.gov/protocoldevelopment/electronic_applications/docs/CTCAE_4.03.xlsx). From the start of radiotherapy until two weeks after the end of chemoradiotherapy, weekly complete blood count and liver function tests were performed. Serum carbohydrate antigen 19–9 (CA19-9) concentration before treatment was evaluated after biliary drainage.

### Statistics

We assumed that the R0 resection rate for BRPC-A patients after neoadjuvant therapy would be 10% to 30%. Our null hypothesis was that the R0 resection rate for those BRPC-A patients confirmed by a radiology would be < 10%. In the current trial, the proposed sample size was 40 patients, which was calculated according to the expected R0 resection rate of 30%, a threshold of 10% and an alpha error of 0.05, with a beta error of 0.05. We expected that 90% of the enrolled patients would start NACIMRT as appropriate, so we decided that the actual sample size should be 45 patients. The final dataset was carefully assessed for clerical errors by three physicians (T.M., K.N., and T.A.). The primary endpoint and the secondary endpoints of the response rate, pathological response, R0 resection rate, surgical morbidity rate, acute and late toxicity of chemoradiation, DFS and OS were evaluated 6 months after the completion of enrollment. Data for continuous variables are expressed as median and range. Kaplan–Meier curves were created to estimate OS, and comparisons between groups were estimated using log-rank tests. To identify risk factors independently associated with survivals, multivariate Cox proportional hazards regression analysis was used. Values of p < 0.05 were considered significant. All statistical analyses were performed with JMP version 15.0 software (SAS Institute, Cary, NC).

## Results

### Patient characteristics

In total, 49 patients were enrolled between June 14, 2013 and March 16, 2021. Baseline characteristics of the cohort are summarized in Table 1. Thirty-four patients showed tumor involvement of the SMA (34/49, 69.3%), while 9 patients showed involvement of the CHA. Thirty-two patients (32/49, 65.3%) had tumors located in the head of the pancreas. PV occlusion or deformation was observed in 28 patients (28/49, 57.1%). Median tumor size was 25.7 mm and median carcinoembryonic antigen (CEA) was within the normal range (< 5.0 U/ml), while median CA19-9 level was 111.4 U/ml. Median maximum standardized uptake value (SUVmax) from FDG-PET was 6.4 and median neutrophil-to-lymphocyte ratio (NL ratio) before neoadjuvant treatment was 2.55.

**Table 1:** Patient Characteristics before NAC

Pre NACIMRT (at enrollment)	*n* = 49
age (years)	68.6(46.0–77.8)
gender (male/female)	29/20
head/body-tail	32/17
radiographic arterial involvement	
celiac artery	4
common hepatic artery	9
replaced RHA	2
superior mesenteric artery	34
BRPV/without BRPV	28/21
radiological tumor size (mm)	25.7(15.0–47.0)
CEA before NACIMRT (U/ml)	3.0(0.5–114.9)
CA19-9 before NACIMRT (U/ml)	111.4(0.6–2451)
NL ratio before NACIMRT	2.55(1.0–10.0)
SUV max before NACIMRT	6.4(2.2–15.8)
NAC incompletion due to adverse event	2/49(4.1%)
Post NACIMRT (at re-assessment after NACIMRT)	*n* = 47
radiological tumor size after NACIMRT (mm)	21.5(11.7–50.1)
CEA after NACIMRT (U/ml)	3.0(0.7–57.6)
CA19-9 after NACIMRT (U/ml)	36.7(0.6–2298)
NL ratio after NACIMRT	3.04(0.81–11.86)
SUV max after NACIMRT	4.35(1.9–10.4)
RECIST > PR	13/47(27.6%)
G3/4 adverse event	10/47(21.3%)

*Abbreviations*: *NAC* neoadjuvant therapy, *IMRT* Intensity Modulated Radiation Therapy, *RHA* right hepatic artery, *BRPV* borderline resectable pancreatic cancer with portal vein involvement, *CEA* carcinoembryonic antigen, *CA19-9* carbohydrate antigen 19–9, *NL* ratio, neutrophil to lymphocyte ratio, *SUV* max, maximum standard uptake value

In the 30 patients who had biliary obstruction, biliary drainage was performed by biliary stenting with a metallic stent in 28 patients (93%) and plastic stent in 2 patients (7%),

### Safety and Clinical outcomes of NACIMRT

**Fig. 2:**
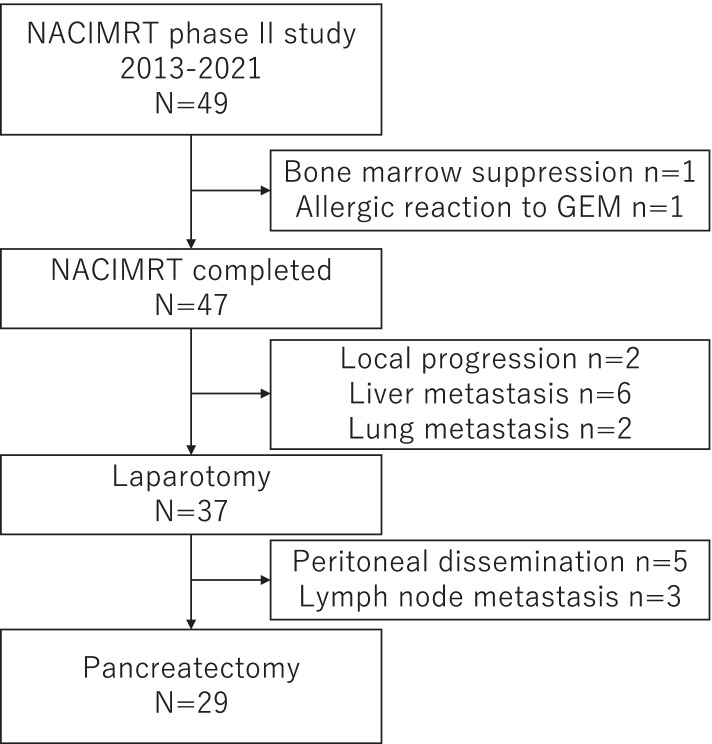
Flow diagram of a phase 2 study with neoadjuvant IMRT. Forty-seven patients (95.9%) completed NACIMRT with gemcitabine and 55.1% (29/49) of the patients underwent pancreatectomy

The study diagram is shown in Fig. 2. The median time from the staging MDCT to the start of the neoadjuvant induction chemotherapy with gemcitabine was 13 days (range, 2–26). Of the 49 patients for whom IMRT was initiated, 47 patients completed IMRT with gemcitabine (95.9%, 47/49). Two patients dropped out due to severe bone marrow suppression and an allergic reaction to gemcitabine. Of these, one patient underwent upfront surgery and the other completed IMRT with S1. The median relative dose intensity of Gemcitabine was 100% and the median total radiation dose was 42 Gy. The preoperative therapy was well-tolerated by all the patients. The frequency of grade 3/4 toxicity in the patients who were initiated on NACIMRT was 24.4% (12/49). The adverse events are listed in Table 2.

**Table 2:** Adverse Events^*^Related to Neoadjuvant gemcitabine and Concurrent IMRT (*N* = 47)

Adverse event	Grade 1–2, *n* (%)	Grade 3, *n* (%)	Grade 4–5, *n* (%)
White blood cell decreased	42(86%)	1(2%)	0(0%)
Neutrophil count decreased	28(57%)	1(2%)	0(0%)
Anemia	38(76%)	0(0%)	0(0%)
Platelet count decreased	30(61%)	1(2%)	0(0%)
Blood bilirubin increased	14(29%)	5(10%)	0(0%)
AST increased	22(45%)	5(10%)	0(0%)
ALT increased	25(51%)	6(12%)	0(0%)
Hypoalbuminemia	32(65%)	1(2%)	0(0%)
Serum AMY increased	10(20%)	4(8%)	0(0%)
Allergic reaction	3(6%)	1(2%)	0(0%)
Fatigue	20(41%)	0(0%)	-
Anorexia	24(49%)	0(0%)	0(0%)
Diarrhea	5(11%)	0(0%)	0(0%)
Mucositis/stomatitis	2(4%)	0(0%)	0(0%)
Nausea	22(45%)	0(0%)	-
Vomiting	8(16%)	0(0%)	0(0%)
Febrile neutropenia	-	0(0%)	0(0%)
Biliary tract infection	-	8(16%)	0(0%)

^*^Events were graded according to Common Terminology Criteria for Adverse Events (CTCAE) version 4.0. *Abbreviations*: *IMRT* intensity modulated radiotherapy, *AST* aspartate transaminase, *ALT* alanine transaminase, *ALB* albumin, *AMY* amylase

In the group that completed NACIMRT, two patients showed local progression and 8 patients had distant metastasis; these 10 patients did not undergo surgical resection. Median CA19-9 concentration decreased from 111.4 U/ml to 36.7 U/ml in patients after completion of NACIMRT. In contrast, NL ratio increased from 2.55 to 3.04 after NACIMRT. The objective radiological response rate was 20.3% and the median radiographic tumor size reduced from 25.7 mm to 21.5 mm. However, radiographic detachment from the major artery after NACIMRT was observed in only 5 patients (10.6%).

### Surgical outcomes and pathological effects

The median interval from completion of IMRT to surgery was 36 days (range, 28–44 days). In the 37 patients who underwent surgery, 5 patients had positive washing cytology and 3 patients had positive distant lymph node metastasis in the para-aortic region at laparotomy, resulting in 29 patients (59.2%) with pancreatectomy.

These 29 patients underwent pancreatectomy with curative intent, and R0 resection was achieved in 27 patients (93.1%) (Table 3). The overall R0 resection rate in the whole cohort was 55.1% (27/49). According to the Evans classification, which pathologically estimates therapeutic effect, complete destruction of the tumor was observed in 1 patient, and > 90% destruction was observed in 1 patient, while < 50% destruction was observed in 10 patients (34.4%). Pathological lymph node metastasis was observed in 10 patients (34.4%).

**Table 3:** Surgical outcomes and Pathological features

Surgical outcome	NACIMRT (*n* = 29)
Type of procedure (PD/DP/TP)	24/4/1
Operation time (min)	552 (370–747)
Blood loss (ml)	558 (125–2900)
PV/SMV resection	21(72.4%)
CHA/SMA resection	4 (13.8%)
CR-POPF (grade B or C)	5 (17.2%)
Clavian Dindo IIIa <	4 (13.7%)
re-operation	0
Duration of in-hospital stay	26.5 (15–58)
in-hospital death	0
Alb 1 month after resection (mg/dl)	3.3 (2.3–4.1)
ChE 1 month after resection (U/L)	186 (77–349)
relative dose intensity of adjuvant S1 (%)	75 (0–100)^a^
Pathological features	
Tumor diameter (mm)	22 (0–55)
R0	27/29 (93.1%)
positive LN metastasis	10 (34.4%)
Evans Grading (1/2a/2b/3/4)	1/9/14/1/1

^a^not started in two patients (judged by a doctor)

*Abbreviations*: *PD* pancreaticoduodenectomy, *DP* distal pancreatectomy, *TP* total pancreatectomy, *PV* portal vein, *SMV* superior mesenteric vein, *CHA* common hepatic artery, *SMA* superior mesenteric artery, *CR-POPF* clinically relevant postoperative pancreatic fistula, *Alb* albumin, *ChE* cholinesterase, *R0* microscopically margin-negative resection, *LN* lymph node

Postoperative grade 3/4 adverse events were observed in 6 patients with 5 patients of clinically relevant postoperative pancreatic fistula (CR-POPF), but no re-operations or in-hospital deaths were encountered. Median duration of the in-hospital stay after resection was 26.5 days.

Median values of both Albumin (Alb) and Cholinesterase (ChE) at 1 month after surgery were within normal ranges in the 29 patients of the eligible cohort. In the 29 patients with R0/1 resection in the cohort, 27 patients (93.1%) started postoperative adjuvant therapy with S1 within 6 weeks after resection. Median relative dose intensity of S1 doses was 75%.

### Survivals

**Fig. 3:**
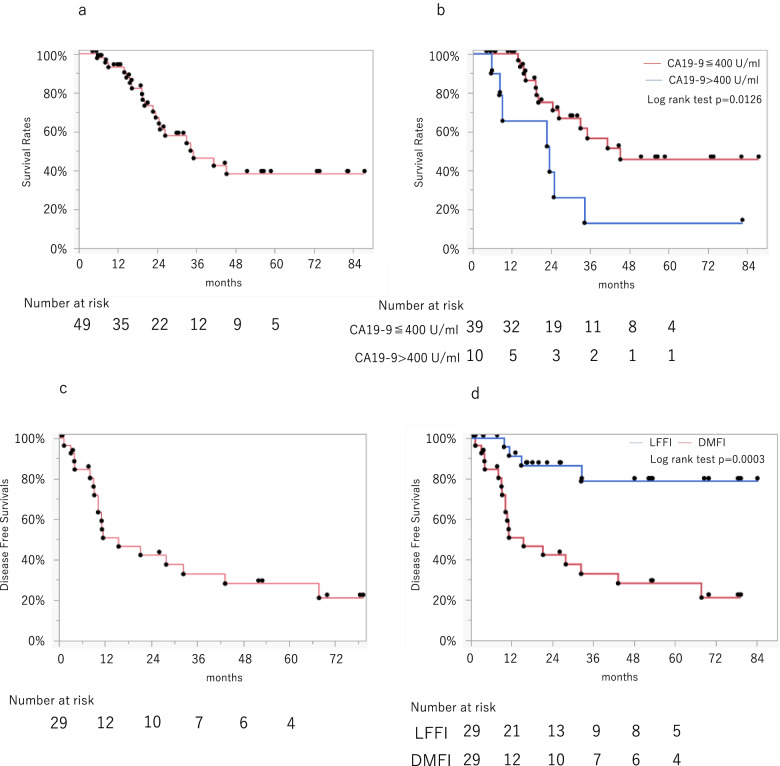
Intention-to-treat analyses of **A**) overall survival in 49 patients with BR-A pancreatic cancer and **B**) overall survival according to CA19-9 at enrollment. Patients with CA19-9 > 400 U/ml showed significantly worse survivals (*p* = 0.0126). **C**) Disease-free survival (DFS) and **D**) locoregional failure-free interval (LFFI) in comparison with distant metastasis-free interval (DMFI). DMFI was significantly worse than LFFI, and comparable to DFS

Kaplan-Meyer plots for survivals are shown in Fig. 3. Median follow-up for the censored patients was 21.0 months. Median OS in the whole cohort (intention to treat) was 35.1 months. (Fig. 3a) Patients with CA19-9 > 400 U/ml at enrollment showed significantly worse survival in this cohort (Fig. 3b). Median DFS of the eligible cohort was 15.5 months and median metastasis-free survival was 15.5 months, while median locoregional failure-free interval could not be calculated because survival rates were over 50% during the observation period (Fig. 3c and d), suggesting a high contribution of distant metastasis to recurrence. To elucidate factors relating to prognosis before treatment, we analyzed pre-NACIMRT factors affecting survivals and identified serum CA19-9 > 400 U/ml as a factor independently associated with overall survival (Table 4).

**Table 4:** Factors at Enrollment associated with Overall Survivals

Variables	Univariate analysis			Multivariate analysis		
	Odds ratio	95%CI	*p* value	Odds ratio	95%CI	*p* value
age (per 1 year)	1.02	0.96–1.10	0.590			
male (v.s. female)	0.79	0.32–1.94	0.608			
head (v.s.body and tail)	0.75	0.30–1.83	0.520			
CA19-9 > 400 U/ml at enrollment	3.08	1.22–7.79	0.018	2.52	1.05–6.63	0.043
diameter > 30 mm at enrollment	1.85	0.76–4.48	0.175	1.78	0.72–4.41	0.212
BRPV at enrollment (v.s. without BRPV)	1.79	0.59–5.36	0.301			
NL at enrollment (per ratio)	1.32	0.79–1.13	0.200	1.24	0.52–1.19	0.297

*Abbreviations*: *CA19-9* carbohydrate antigen 19–9, *BRPV* borderline resectable pancreatic cancer with portal vein involvement, *NL* neutrophil to leukocyte ratio

## Discussion

BRPC is rare compared to resectable or unresectable PC, following difficulty in analyzing large series. Nagakawa et al. recently presented a large, retrospective study of BRPC with propensity-matched analyses to elucidate the effects of neoadjuvant radiation in 272 patients, revealing the R0 rate to be 87.2% in the neoadjuvant radiotherapy group [19]. However, total resectability was unclear because of the retrospective design.

The current phase 2 prospective study investigated the safety and the efficacy of IMRT with concurrent gemcitabine in 49 BRPC-A patients. In this cohort, 47 out of 49 patients completed NACIMRT, there was no Grade 4 or 5 adverse events observed. Although there were 8 patients who suffered bile duct infection, all of these patients recovered with antibiotics. In terms of efficacy, R0 was completed in 93% of resected patients (27/29), and 55.1% of initial BRPC-A patients (27/49) underwent curative resection. Our previous phase 2 study using gemcitabine and S1 as a neoadjuvant therapy for BRPC-A patients (NACGS study) showed that 73% of R0 resections and 60.8% of initial BRPC-A patients completed curative pancreatectomy, broadly comparable to the current study. However, for OS and PFS, our current NACIMRT study showed favorable survival compared to our previous NACGS study (NACIMRT vs. NACGS; median OS: 35.1 months vs. 21.7 months, median PFS: 15.5 months vs. 13.9 months) [8].

In comparison to other prospective trials, the PREOPANC study, a randomized phase 3 study for Resectable (R) and Borderline Resectable (BR) pancreatic cancer with neoadjuvant therapy, showed a good R0 rate (NAC 79% versus upfront 13%) but low resection rate (NAC 52% versus upfront 64%) in the subclass analysis with BRPC (*n* = 113), compared to immediate surgery [6]. Jang et al. compared NACRT and NAC in a BRPC with Randomized Control Trial (RCT) study (*n* = 50) and also found a high R0 rate with NACRT (82.4%) compared to NAC (33.3%), while resection was performed in 62.9% (17/27) of NACRT patients, resulting in a 34% resection rate [7]. The recent JASPAC05 phase 2 trial found an R0 resection rate of 74% (29/39), while resection was performed in 55.7% (29/52) [20] for BRPC. Although patients enrolled in the current study were limited to BRPC with artery abutment, our study also revealed a high R0 rate with a fair resection rate. The low resection rate was mainly attributed to distant metastases, as 16 of 18 patients were found to have distant metastasis before pancreatectomy. In turn, this indicates preferable control of local progression by NACIMRT. Indeed, our locoregional failure-free intervals were significantly better than distant metastasis-free intervals after resection.

Despite the high R0 status and low resectability in the current study, as in previous studies, our current DFS and OS showed relatively high rates of long-term survivors compared to other NACRT studies for BR. Five-year survival rates were 28.2% for DFS and 38.3% for OS in the whole cohort. One important issue for this preferable outcome was that we applied IMRT, which can simultaneously reduce the dose to surrounding normal organs, while assuring adequate target dose coverage compared to conventional RT techniques. A recent study suggested that a customized clinical target volume that specifically includes the SMA and CA will improve coverage to this region and will account for individual and tumor variability [21]. The present series included the region around the SMA and CA in the CTV in addition to the GTV. In addition, we applied a hypofractionated dose of 42 Gy in 15 fractions, of which the biological dose is almost equivalent to the conventional standard treatment dose (48.6–50.4 Gy in fractions of 1.8 Gy). We did not intend to escalate the delivered dose because this study is a preoperative setting. The purpose of hypofractionated IMRT is to reduce the gastrointestinal toxicities with shortened radiation treatment period.

We have previously reported favorable outcomes of hypofractionated IMRT on Locally Advanced Pancreatic Cancer (LAPC) in elderly patients without severe toxicities [22]. One possibility is that current hypofractionated IMRT contributed to a high rate of S-1 introduction (93.1%, 27/29) and a high relative dose intensity of S1 (median, 75%) for adjuvant treatment of BRPC in post-NAC patients. Indeed, a high relative dose intensity of adjuvant S1 therapy in BRPC patients treated by IMRT was reported in comparison to patients treated by standard CRT [23]. In the JASPAC05 study, a neoadjuvant trial of S-1 combined with conventional CRT, a lower 82% (22/27) of patients than in the present study were reported to receive adjuvant therapy after surgery, but the protocol for adjuvant therapy was not defined [20]. This might be another reason for the favorable outcomes in our study. We verified the daily patient position using CBCT, but since huge advances in the technology such as hybrid MRI-Linac have been recently introduced that can help to deliver treatments in breath-hold irradiation, more accurate radiation treatment will be possible in the near future. [24].

In the current analysis evaluating pretreatment factors associated with survival, initial serum CA19-9 > 400 U/ml was an independent risk factor for overall survival, irrespective of tumor size. A reduction in serum CA19-9 or normalized CA19-9 after NAC is reportedly associated with good prognosis [25, 26], but categorizing those patients for whom neoadjuvant treatment should be applied beforehand, in addition to anatomical status, is difficult. Recently, high serum CA19-9 has been proposed as a “biological BR” in pancreatic cancer which shows worse survivals [27] and some recommend neoadjuvant treatment for such patients [28]. At the very least, the current study revealed that higher serum CA19-9 remains a worse prognostic factor, even with NACIMRT. Considering the high recurrence rate in terms of distant metastasis in this cohort, other modalities such as strong systemic chemotherapy aiming for reduction of micrometastases should be examined in combination with IMRT.

This study has several limitations. First, this was a study at a single institution. Although IMRT potentially reduces the toxicities of RT to normal organs with adequate target dose coverage, the GTV and CTV should be planned according to individual tumor location, spread and vascular anatomies in a standardized procedure. Standardizing the outcomes of IMRT in a multi-institutional study is thus difficult in terms of variety of quality. Second, the current enrolled BRPC patients were limited to patients with BRPC showing arterial abutment. Because a low R0 rate was most frequent in BRPC-A, we focused on this category of patients. The current study showed that local control was favorable even in BRPC-A, in which curative resection is more difficult and recurrence occurs more frequently at distant sites, suggesting that BRPC including BRPC-V and BRPC-A should be treated with the main focus on distant metastasis. Third, because this was not a randomized clinical trial, comparison with outcomes from other modalities such as FOLFIRINOX or gemcitabine/ nab paclitaxel is difficult.

## Conclusions

The current GEM based NACIMRT phase 2 study showed a resectability rate of 55.1% for BRPC-A patients, who initially showed tumor abutment to major arteries. Median OS for the entire cohort was 35.1 months and the 5-year OS rate was 38.3% with good feasibility of neoadjuvant IMRT with concurrent gemcitabine. As initial low CA19-9 is important for long survival with NACIMRT treatment, patients with high CA19-9 may need another strategy.

## Data Availability

The datasets used and/or analysed during the current study are available from the corresponding author on reasonable request.

## Abbreviations

BRPC: Borderline resectable pancreatic cancer
IMRT: Intensity-modulated radiation therapy
NACIMRT: Neoadjuvant chemotherapy using IMRT
BRPC-A: Borderline resectable pancreatic cancer with arterial abutment
R0: Microscopically margin-negative resection
CA19-9: Carbohydrate antigen 19–9
NCCN: The National Comprehensive Cancer Network
SMA: Superior mesenteric artery
CA: Celiac axis
CHA: Common hepatic artery
PHA: Proper hepatic artery
SMV: Superior mesenteric vein
PV: Portal vein
MDCT: Multidetector-row computed tomography
FDG-PET: Positron emission tomography with 2-deoxy-2-[^18^F]fluoro-D-glucose
MRI: Magnetic resonance imaging
EOB-MRI: MRI with contrast medium of gadolinium-ethoxybenzyl diethylenetriaminepentaacetic acid
GTV: Gross tumor volume
CTV: Clinical target volume
PTV: Planning target volume
LFFI: Locoregional failure-free interval
DMFI: Distant metastasis-free interval
DFS: Disease-free survival
OS: Overall survival
SUVmax: Maximum standardized uptake value
CEA: Carcinoembryonic antigen
NL ratio: Neutrophil-to-lymphocyte ratio
CR-POPF: Clinically relevant postoperative pancreatic fistula
Alb: Albumin
ChE: Cholinesterase
LAPC: Locally advanced pancreatic cancer
